# A latent class analysis of resilience and its association with patient-reported symptoms in patients with esophageal cancer after esophagectomy

**DOI:** 10.3389/fpsyg.2023.1241129

**Published:** 2023-10-19

**Authors:** Yanran Li, Zonghao Zhang, Xuanxuan Ma, Xue Zhang, Shuwen Li

**Affiliations:** Department of Nursing, Anhui Medical University, Hefei, China

**Keywords:** resilience, esophageal cancer, latent class analysis, patient-reported, symptom

## Abstract

**Purpose:**

To identify the latent classes of resilience in patients with esophageal cancer after esophagectomy and develop a deeper understanding of the association between these classes and patient-reported symptoms.

**Background:**

China accounts for more than half of the global burden of esophageal cancer, and patients with esophageal cancer experience numerous symptoms that affect their quality of life and prognosis. Given that resilience is a key element that alleviates the progression of symptoms, it may represent a potential means of to enhancing cancer patients’ physical and psychological well-being.

**Methods:**

The study was implemented in the thoracic surgery departments of three tertiary hospitals in eastern China. The participants were patients who were still hospitalized after esophagectomy. Data were gathered by self-report questionnaires, and a latent class analysis was utilized to identify different categories of resilience among the patients.

**Results:**

A total of 226 patients were recruited. The three classes of resilience identified included high strength and striving (53.5%), medium resilience but weak self-recovery (35.9%), and minimal tenacity and external support (10.6%). Patients with low income (OR = 12.540, *p* = 0.004) were more likely to be in the minimal tenacity and external support class. Patients without comorbidities (OR = 2.413, *p* = 0.013) and aged 66–70 years (OR = 4.272, *p* < 0.001) were more likely to be in the high strength and striving class. The patient-reported symptoms and symptom-related interference of patients after esophagectomy varied considerably among the three categories of resilience.

**Conclusion:**

Accurate interventions should be devised and executed according to the features of each type of resilience in patients after esophagectomy to maximize intervention efficacy. These findings highlight the important role of precision nursing.

## Introduction

Esophageal cancer has impacted an increasing number of individuals worldwide, with research estimating 604,000 new cases and 544,000 fatalities in 2020 (Sung et al., 2021). Esophageal cancer is concentrated in specific regions, with 346,633 new cases (over half of the global average)expected in China in 2022 (Xia et al., 2022). Although China’s esophageal cancer mortality rate is falling, the relative five-year survival is just 30.3%, which is low (He et al., 2021). Surgery is a common and efficient treatment for esophageal cancer. Due to both the cancer and treatment, after esophagectomy patients experience multiple symptoms, such as lack of appetite, difficulty swallowing, nausea/vomiting, acid reflux, cough/dry mouth, pain, fatigue, distress, shortness of breath, and disturbed sleep (Guo et al., 2019; Gupta et al., 2020). These symptoms are frequent, with an incidence ranging from 54.2 to 83.9% (Wang et al., 2022). Given that the symptoms experienced by patients after esophagectomy could significantly affect their quality of life and prognosis (Liu et al., 2021; Wang et al., 2022), it is imperative to investigate available interventional elements that could lessen the likelihood of these symptoms or alleviate symptoms.

Resilience is an evolving concept that includes the ability of people to acquire resources to successfully cope with challenges and encourage recovery from negative events (Stainton et al., 2019). In the growing research area of cancer care research area, resilience has attracted increasing interest because the consequences of resilience may influence patient functioning (physical, psychological, and social functioning) (Luo et al., 2020), and resilience is strongly associated with the intensity and/or absence of symptoms (Tamura et al., 2021). For patients with esophageal cancer, those with higher resilience report less symptom distress (Guo et al., 2019). Therefore, increasing the level of resilience is potentially represents a viable solution to alleviate self-reported symptom and symptoms distress for among patients after esophagectomy.

Until recently, the primary method for evaluating the levels of resilience in adult cancer patients has been to calculate a total scale score (Sihvola et al., 2022), which is a variable-centered approach and overlooks the heterogeneity among individuals within the group. Several studies have used person-centered techniques to measure resilience according to individuals’ characteristics, such as in breast cancer patients (Ye et al., 2021) and parents of children with cancer (Luo et al., 2022), and the findings suggest that there are significant individual differences in resilience among various subgroups. However, patients with each types of cancer have different treatment modalities, symptom experiences, recovery processes, and clinical outcomes, and specific resilience manifestations are mutable. Therefore further validation in the esophageal cancer population is warranted. Tenacity, strength, and optimism are the three components of resilience, According to the conservation of resources theory (Bouckenooghe and Raja, 2019), strength and optimism are gain-oriented resources, while tenacity is a loss-oriented resources, this also suggests that there might be different categories of resilience.

Latent class analysis (LCA), a person-centered technique, classifies individuals into latent classes depending upon their similarities and differences across investigated features to better understand patterns in the population. LCA can be used to explore the heterogeneity of resilience in patients with esophageal cancer because of its ability to identify heterogeneous patient groups, similar to its earlier successful application in other adult cancer patients (Malgaroli et al., 2022; Wallstrom et al., 2022). With the development of precision nursing (Harrington, 2021), there is increasing interest in tailoring supportive care according to each patient’s unique characteristics and requirements, which would ultimately maximize the effect of interventions and help achieve the best clinical outcomes. Therefore, it is necessary to evaluate the characteristics of resilience among patients after esophagectomy and assessing their health requirements to develop specialized resilience interventions, such as intervention programs based on the protective factors of resilience.

Therefore, the current study’s objectives were as follows: (1) to identify different classes of resilience in patients after esophagectomy using LCA; (2) to recognize sociodemographic and clinical characteristics that distinguish individuals with different classes of resilience, and (3) compare the differences in patient-reported symptoms between each class of resilience to develop targeted resilient interventions.

## Methods

### Participants and procedure

A cross-sectional investigation was performed at the thoracic surgery department of three tertiary hospitals in Anhui Province, eastern China, from May 2021 to August 2022. The inclusion criteria for patients were as follows: (1) pathologically diagnosed with stages I to III esophageal cancer, hospitalized and underwent esophagectomy; (2) Chinese speaker with a minimum age of 18 years; (3) able to complete the questionnaire; (4) free of mental disorders. Those with recurrence after radiotherapy or chemotherapy were excluded. Two trained research nurses from each of the three hospitals were assigned to the responsible ward to carry out the acquisition of data. All eligible patients who underwent surgery for esophageal cancer received invitations to participate in the study during the survey period. The designated study nurse contacted patients and offered them a self-report paper questionnaire if they agreed to participate; this questionnaire, this questionnaire, took the patients approximately 15–20 min to complete.

### Sample size and estimated study power

**Figure 1:**
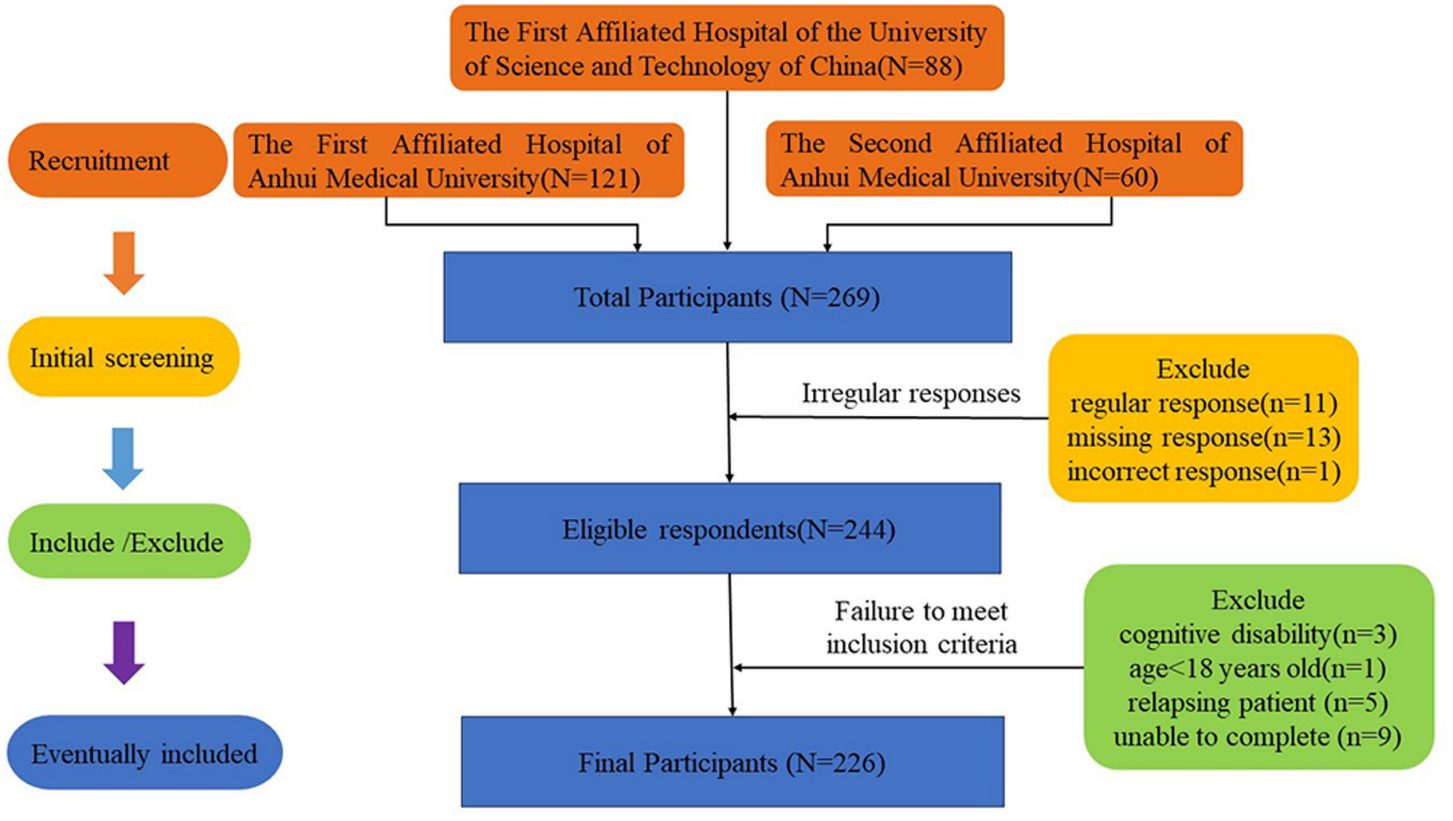
Flow diagram of patient inclusion and exclusion.

According to sample sizes calculated for the bootstrap likelihood ratio test in LCA (Dziak et al., 2014), a minimum sample size of 220 was needed in this investigation to generate a power of 0.8 at a 0.05 significance level. A total of 269 patients agreed to participate in this study, of whom 18 who did not meet the inclusion criteria and 25 with irregular questionnaire responses were excluded. Finally, 226 patients were included in the data analysis. See Figure 1 for the specific inclusion and exclusion criteria.

### Measures

#### Demographic and clinical data

The participants’ demographic and clinical characteristics were collected with a basic information worksheet. Sex, age, marital status, years of education, location of residence, work status, and monthly family income were among the demographic details. Clinical information included the focal site of cancer, surgical approach, combined chronic disease, frequency of hospital admission.

#### Connor-Davidson resilience scale

The CD-RISC, a self-report psychological resilience scale, was developed by Connor and Davidson (2003)). A Chinese version of the scale was sinicized and tested in a local population over the age of 18 years by Yu and Zhang (2007)). Three dimensions—tenacity, strength, and optimism—are each represented in the 25 items. Items are scored on a 5-point Likert scale (0–4): not true whatsoever (0), seldom true (1), occasionally true (2), frequently true (3), and virtually always true (4). Because of its high degree of reliability and validity, the scale has been frequently employed in medical institutions (Boskailo et al., 2021). The internal consistency coefficient of the Chinese version of the CD-RISC was 0.91. The internal consistency alpha values of the 3 factors were 0.88 for tenacity, 0.80 for strength, and 0.60 for optimism. In this study, the Cronbach’s alpha of this scale was 0.89.

#### MD Anderson symptom inventory for gastrointestinal cancer

The MDASI-GI, a module of a symptom self-assessment scale specific to gastrointestinal cancer was developed by the MD Anderson Cancer Center (Wang et al., 2010). The Chinese version was translated and tested in individuals with advanced or postoperative gastrointestinal cancer (Chen et al., 2019). It is divided into two sections: the first includes 13-items on core symptoms and 5-items on gastrointestinal cancer-specific symptoms; that severity of symptoms is evaluated within the past 24 h, of 0 to 10 representing a transition from “hardly anyhow” to “as awful as you can envisage,” and a higher rating reflects higher severity of symptoms. The second section, consisting of six items, assesses symptom-related interference over the same period, and the items are similarly rated on a scale ranging from 0 to 10 (0 = “absence of invasion” to 10 = “fully invasion”). The total Cronbach’s alpha coefficient for all symptom items was 0.89. For the symptom severity section, the Cronbach’s α was 0.82, and for the interference magnitude, it was 0.87.

### Data analysis

LCA was carried out via Mplus v 7.0 software to identify heterogeneous subgroups of resilience in esophageal cancer patients. First, the scores for each CD-RISC item were dichotomized into 0 or 1. An original score ≥ 4 was considered to have a high response probability and was scored as 1, while an original score < 4 was considered as a low response probability and was scored as 0. The converted data were then input into potential class analysis, and several models—from the inaugural one class to the final four classes—were calculated by progressively increasing the amount of classification when the best-fit indices were reached. Six model fit metrics, the Akaike information criterion (AIC), Bayesian information criterion (BIC), adjusted Bayesian information criterion (aBIC), Lo-Mendell-Rubin (LMR), bootstrap likelihood ratio test (BLRT), and entropy, were used to identify the optimal model. The AIC, BIC and aBIC are commonly used to compare different pairs of models, with the lowest value on each indicator indicating the best fit. The LMR and BLRT are used to compare the estimated model with a model containing k-1 class(es), where k equals the number of classes. Entropy is used to assess the accuracy of model classification, which is usually considered better good if Entropy >0.8. IBM SPSS Statistics 25.0 was employed for other data analyses. The mean and standard deviation (SD) are used to report continuous variables, whereas the frequency and percentage are chosen to depict categorical variables.

After the best model was chosen, a multinomial regression analysis was performed to investigate the elements impacting the different categories of resilience regarding the demographic and clinical parameters. Finally, the symptoms of patients who belonged to each category of resilience were compared using one-way analysis of variance (ANOVA), and a *post hoc* test was performed, using Bonferroni correction if the variance was homogeneous and Tamhane T2 if the variance was not homogeneous.

### Ethical considerations

This study was approved by the Biomedical Ethics Committee of Anhui Medical University (Ref no. 2021H011). The inquiry was carried out in line with the most recent version of the Helsinki Declaration. After the patients consent to be enrolled in this study, they signed written informed permission that included special details concerning the study’s procedures. All participants had the ability to back out of the research at any point, and their health care and entitlements were unaffected.

## Results

### Participant characteristics

A total of 226 individuals were include, the majority of whom were married (93.8%) and male (72.6%), had an average age of 68.7 years (SD = 8.6, range 45–92), reported primary or lower education (61.5%), resided in rural areas (61.5%), and had a family income of less than 3,000 CNY per month (55.8%). The majority of patients had esophageal cancer sites in the middle and lower regions (83.2%), and had undergone minimally invasive surgery (87.6%) and were hospitalized for the first time (89.4), with 55.8% having no comorbidities. Table 1 shows the attributes of the patients who underwent surgery for esophageal cancer.

**Table 1:** Participant attributes.

Characteristics	Frequency	%
**Sex**		
Male	164	72.6
Female	62	27.4
**Age**		
≤65	75	33.2
66–70	52	23.0
≥71	99	43.8
**Marital status**		
Married	212	93.8
Widowed	11	4.90
Single	3	1.30
**Education status**		
Primary or none	139	61.5
Secondary	52	23.0
Tertiary or above	35	15.5
**Work status**		
Farmer	140	61.9
Retiree	44	19.5
Worker	42	18.6
**Residential status**		
Rural	139	61.5
County	54	23.9
Urban	33	14.6
**Family’s earning per month** (**¥, CNY**^**a**^)		
<3,000	126	55.8
3,000 ~ 4,999	69	30.5
≥5,000	28	12.4
Missing	3	1.30
**Treatment**		
Minimally	198	87.6
Open esophagectomy	28	12.4
**Tumor location**		
Upper	38	16.8
Middle	121	53.5
Lower	67	29.6
**Number of hospitalizations due to EC**		
1 time	202	89.4
2 or more times	24	10.6
**Comorbidity**		
No	126	55.8
Yes	100	44.2

a CNY China Yuan, US$ 1.00 = ¥ 6.91.

### Latent classes of resilience

In LCA, one model was first investigated, followed by models with progressive increases in the number of classes in investigated a sequential manner, and four models were finally analyzed. The three-class model was settled on the best-fitting model because of its low AIC, BIC, and aBIC values, substantial LMR-LRT and BLRT *p* values, and high entropy value of 0.963. The LMR-LRT *p* value did not significantly differ between the four-class model and three-class models. Table 2 displays the model fit indices.

**Table 2:** Model fit indices of different classes of resilience.

Model	Log(L)	AIC	BIC	aBIC	LMR-LRT *p* value	BLRT *p* value	Entropy
1	−3715.74	7481.48	7566.99	7487.76	–	–	–
2	−2951.99	6005.99	6180.44	6018.81	<0.001	<0.001	0.966
3	−2858.75	5871.50	6134.88	5890.85	0.0006	<0.001	0.963
4	−2794.62	5795.24	6147.56	5821.13	0.355	<0.001	0.910

Log(L), log likelihood; AIC, Akaike Information Criterion; BIC, Bayesian Information Criterion; aBIC, adjusted Bayesian Information Criterion; LMR-LRT, Lo-Mendell-Rubin Likelihood Ratio Test; BLRT, Bootstrap Likelihood Ratio Test. –, not applicable.

**Figure 2:**
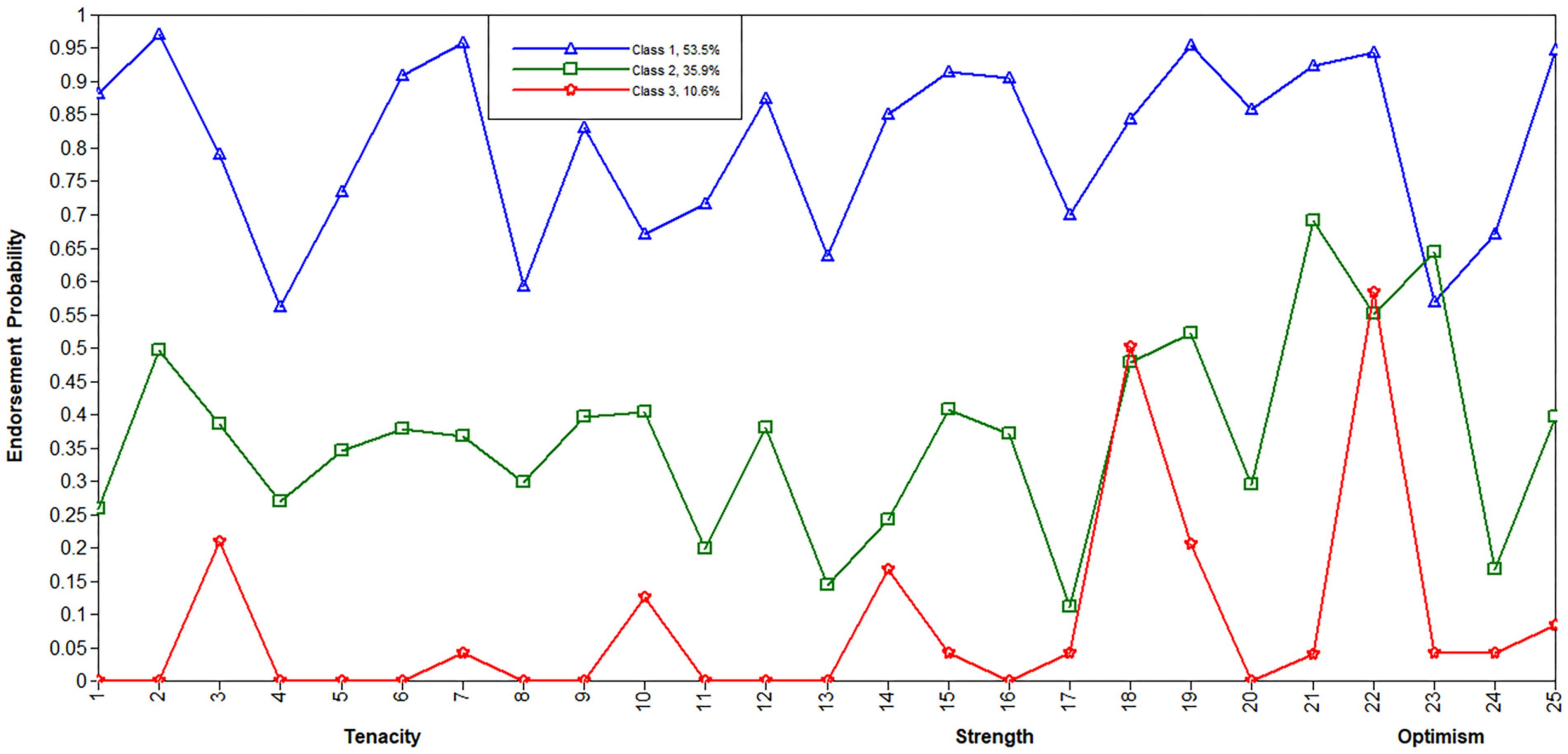
Overview of the 3-class LCA model of resilience. The x-axis denotes resilience items, while the Y-axis shows each item’s endorsement probability. All resilience items were realigned in dimensions, with Items 1–13 reflecting tenacity, 14–21 reflecting strength, and 22–25 reflecting optimism. Class 1, high strength and striving; Class 2, moderate resilience but weak self-recovery; Class 3, minimal tenacity and external support.

The endorsement probability of each item was used to determine each class. According to a prior study (Luo et al., 2022), values above 0.6 were deemed high probability, between values 0.60 and 0.15 were considered moderate probability, and below values 0.15 were considered low probability. As a result, the three classes of resilience patterns were identified: high strength and striving, medium resilience but weak self-recovery, and minimal tenacity and external support. Figure 2 presents the specific responses of patients in of each of the three categories to all 25 CD-RISC items.

Class 1 (high strength and striving, 53.5%) involved considerably higher endorsement probabilities on all three dimensions of resilience, with 0.87 on the strength dimension, 0.79 on the optimism dimension, and 0.78 on the tenacity dimension; the strength dimension had the highest endorsement probability. Notably, this class had multiple highly probable items (>0.90), such as Item 2 (When things look hopeless, I do not give up), Item 7 (Think of self as a strong person), Item 15 (Past success gives confidence for a new challenge), Item 16 (Coping with stress strengthens), and Item 19 (Best effort no matter what). Therefore, this class was dubbed the ‘high strength and striving class’, since these individuals had a strong desire to overcome adversity, and they were able to press forward when faced with difficulties and challenges.

Class 2 (medium resilience but weak self-recovery, 35.9%) acted out moderate endorsement likelihoods on all three dimensions of resilience, with 0.33 on the tenacity dimension, 0.39 on the strength dimension and 0.44 on the optimism dimension, thus, the patients in this class had medium resilience. While, the expression of a couple of low propensity items, for illustration, Item 17 (Tend to bounce back after illness or hardship) had an endorsement probability of 0.11, and Item 24 (Can deal with whatever comes) had an endorsement probability of 0.15, suggesting that the patients had low self-recovery, especially when faced with adversity, and that it was difficult for them to recover.

Class 3 (minimal tenacity and external support, 10.6%) exhibited low probabilities of almost all items on the three dimensions of resilience, with 0.03 on the tenacity dimension, 0.13 on the strength dimension and 0.19 on the optimism dimension. In particular, most items on the tenacity dimension had response probabilities below 0.10, which indicates that the patients in this class had low tenacity and could be incapable of overcoming difficulty. Fortunately, these individuals had moderate probabilities of several items, such as Item 22 (Close and secure relationships) had an endorsement probability of 0.58, item 18 (Things happen for a reason) had an endorsement probability of 0.50, and item 3 (Know where to turn for help) had an endorsement probability of 0.21, indicating that when confronted with adversity or pressure, the patients know where to get help and were confident that they could obtain external support.

### Factors affecting the classes of resilience

To determine the clinical and demographic characteristics that dictated the likelihood that patients would be assigned to the three memberships, a multinomial regression examination was performed to compare the classes. Compared to Class 1, Class 3 was more likely to contain patients with a low income (family’s earning per month < 5,000) (odds ratio [OR] = 12.540, *p* = 0.004). Compared to Class 2, patients without comorbidities (OR = 2.413, *p* = 0.013) and aged 66–70 (OR = 4.272, *p* < 0.001) were more probably to be in class 1. Compared to Class 3, patients with low income (family’s earning per month < 5,000) had a lower probability in class 2 (OR = 0.157, *p* = 0.037). There was no significant variation among the three classes in regard to gender, occupation, residential location, or treatment. Table 3 illustrates the demographic and clinical details that were found to affect memberships in the three of resilience classes.

**Table 3:** Multinomial regression examination for three classes of resilience.

	Variable	*B*	SE	Wald	Exp(B)	95% CI	*p* value
Class 3 vs. Class 1	**Age**						
	≤65	0.408	0.835	0.239	1.504	0.293–7.728	0.577
	66 ~ 70	−0.823	0.581	2.006	0.439	0.140–1.372	0.157
	≥71^a^						
	**Gender**						
	Male	−0.614	0.570	1.161	0.541	0.177–1.653	0.281
	Female						
	**Work status**						
	Retiree	−0.589	1.083	0.295	0.555	0.066–4.639	0.587
	Worker	−1.155	0.913	1.602	0.315	0.053–1.884	0.206
	Farmer^a^						
	**Residential status**						
	Rural	−0.558	1.095	0.260	0.572	0.067–4.896	0.610
	County	0.316	1.081	0.086	1.372	0.165–11.408	0.770
	Urban^a^						
	**Family’s earning per month**						
	**<3,000**	**2.284**	**0.903**	**6.400**	**9.820**	**1.673–57.637**	**0.011** ^*^
	**3,000 ~ 4,999**	**2.529**	**0.874**	**8.363**	**12.540**	**2.259–69.611**	**0.004** ^*^
	≥5000^a^						
	**Treatment**						
	Minimally	−0.226	0.804	0.079	0.798	0.165–3.856	0.779
	Esophagectomy^a^						
	**Comorbidity**						
	No	0.080	0.547	0.021	1.083	0.370–3.167	0.884
	Yes^a^						
Class 1 vs. Class 2	**Age**						
	≤65	0.899	0.565	2.533	2.458	0.812–7.441	0.112
	**66 ~ 70**	**1.452**	**0.387**	**14.067**	**4.272**	**2.000–9.122**	**<0.001** ^*^
	≥71^a^						
	**Comorbidity**						
	**No**	**0.881**	**0.353**	**6.213**	**2.413**	**1.207–4.825**	**0.013** ^*^
	Yes^a^						
Class 2 vs. Class 3	**Family’s earning per month**						
	<3,000	−1.790	0.919	3.795	0.167	0.028–1.011	0.051
	**3,000 ~ 4,999**	**−1.850**	**0.885**	**4.367**	**0.157**	**0.028–0.892**	**0.037** ^*^
	≥5000^a^						

Class 1, high strength and striving; class 2, moderate resilience and weak self-recovery; class 3, low tenacity and external support.

a Reference group.

*p < 0.05.

The bold is to highlight statistically significant data, it means that the data is statistically significant.

### Patient-reported symptoms in each class of resilience

The ANOVA results revealed that the core symptoms and symptom-related interference (MDASI-GI scores) reported by the patients varied considerably among the three memberships of resilience. The *post hoc* test using Bonferroni correction or Tamhane’s T2 method for multiple comparisons demonstrated that the scores on core symptoms (*F* = 22.490, *p* < 0.001) and symptom-related interference (*F* = 34.317, *p* < 0.001) in Class 1 were substantially greater than those in the other two classes. There were no statistically significant variations in gastrointestinal cancer-specific symptoms (*F* = 1.859, *p* = 0.158) among the three latent classes. Table 4 provides a examination of symptoms across each class of resilience.

**Table 4:** Patient-reported symptoms in three dimensions among each class of resilience.

	Core symptoms		Gastrointestinal cancer-specific symptoms		Symptom-related interference	
	ANOVA					
	*F*	*p* value	*F*	*p* value	*F*	*p* value
Variable group	**22.490**	**<0.001** ^*^	1.859	0.158	**34.317**	**<0.001** ^*^
	Pairwise comparisons (mean differences) between classes					
Contrast	Mean difference	*p* value	Mean difference	*p* value	Mean difference	*p* value
Class 3 vs.						
Class 2	**11.423**	**0.038** ^*^	−1.971	0.204	**12.630**	**<0.001** ^*^
Class 1	**22.647**	**<0.001** ^*^	−1.887	0.207	**21.598**	**<0.001** ^*^
Class 2 vs.						
Class 1	**11.224**	**<0.001** ^*^	0.084	0.899	**8.968**	**<0.001** ^*^

Class 1, high strength and striving; class 2, moderate resilience and weak self-recovery; class 3, low tenacity and external support.

*p < 0.05.

The bold is to highlight statistically significant data, it means that the data is statistically significant.

## Discussion

To the best of our knowledge, this is the first study to investigate the characteristics of resilience and their association with self-reported symptoms among patients after esophagectomy. We recognized three categories of resilience that may serve as a starting point for future research, and LCA could also be used to identify intra-class discrepancies.

The participants in the high strength and striving class (Class 1) accounted for 53.5% of the total and exhibited high probabilities of almost all items in the three dimensions of resilience. Patients in this group had strong beliefs and enough tenacity to effectively cope with the cancer; in turn, effective coping made patients stronger and more capable of overcoming new difficulties and adversities (Macía et al., 2021; Karademas et al., 2023). This positive feedback loop enables patients to successfully adjust to life after cancer. Peer support and observation of role models among patients who underwent surgery for esophageal cancer may provide some benefits, such as discussion of effective coping experiences, maintaining a positive mindset, and acknowledging the new situation (Nielsen et al., 2021), which may enable future patients to play a more active role in self-governance and quality of life. This means that directing patients in the high strength and striving class to assist other patients with esophageal cancer would result in a win-win situation.

The medium resilience but weak self-recovery (Class 2), accounting for 35.9% of the participating people, had average odds on the three dimensions of resilience. The items with the lowest probabilities indicated that this group of patients had poor self-recovery capacity, which will make it difficult for them to recover from cancer, and frequently leads to psychological distress such as pessimism or disappointment. Self-efficacy is a crucial factor in determining a person’s potential to recover, and could promote self-management and personal recovery (Ibrahim et al., 2022). Resilience and self-efficacy are inextricably related, for grown cancer patients, self-efficacy plays a substantial role in the concept of resilience and mediates the relationship between resilience and how people cope with illness (Wu et al., 2021; Yin et al., 2022). A recent systematic literature review also manifested that self-efficacy was interrelated to resilience as internal factors (Tamura et al., 2021). A considerable number of studies has demonstrated that comprehensive psychological/resilience intervention programs, such as psychological interventions in the light of PERMA (Positive Emotion, Engagement, Relationships, Meaning, and Accomplishment) framework (Tu et al., 2021), stress management and resilience training (Marzorati et al., 2021), CBT (Cognitive Behavioral Therapy) (van Helmondt et al., 2023), mindfulness-based interventions (Galante et al., 2021), and emotive behavior therapy (Liu et al., 2022), have different degrees of effectiveness on overall resilience and ultimately enhanced physical and psychological well-being. However, most psychological/resilience intervention programs rarely incorporate self-efficacy or have nonsignificant effects on self-efficacy (Jin et al., 2022). A previous study implemented a one-on-one resilience program on the basis of protective factors, which could upgrade both resilience and self-efficacy for in cancer patients (Jiang et al., 2019). Therefore, among patients in the medium resilience but weak self-recovery class, targeted interventions focusing on comprehensive enhancement of resilience and self-efficacy would be more effective.

The minimal tenacity and external support (Class 3) accounted for 10.6% of the participants; these participants had low probabilities of endorsing, the majority of items, particularly those in the tenacity dimension. This group of patients demonstrated a lack of fortitude, composure, and tenacity to overcome hardship after receiving a cancer diagnosis. The items with moderate probabilities, on the other hand, indicated that patients in this group knew where to find support and who could supply it when faced with difficulties. Social support, as one of the essential resilience-boosting psychological and social assets of grown cancer patients, could enhance psychological resilience (Harms et al., 2019), minimize social isolation (Clifton et al., 2022), and physical symptoms (Zeilani et al., 2023), and improve quality of life (Ruiz-Rodriguez et al., 2022). As a result, developing tenacity-focused interventions and encouraging patients to actively seek illness-related practical support would be extremely beneficial for patients in the minimal tenacity and external support class, helping them to effectively respond to the cancer diagnosis.

The multinomial regression results clearly showed that patients with a monthly household income <5,000 (average rural household income in Anhui) were at risk of exhibiting a maladaptive response. This result is in line with the findings of a previously published study that claimed that cancer patients in rural China with lower household incomes had weaker resilience and poor quality of life (Su et al., 2019). Cancer patients, especially those in rural areas, are relatively poor, and the current health insurance system in China does not completely relieve the burden of medical expense (Xia et al., 2023), Excessive medical cost would aggravate patients’ family economic burden, imposing a sense of powerlessness, thereby weakening resilience and leading to varying severity of psychological distress. So, reducing medical expense and increasing the scope of medical assistance and insurance system coverage (Yang et al., 2022), as well as strengthening the accessibility of health care (Wang et al., 2022), may be potentially feasible approaches to reduce the negative impact of poor socioeconomic position. In addition, patients aged 66–70 years exhibited higher levels of resilience and were more likely to be able to cope with their disease successfully. This might be because the majority of the participants (72.6%) were men, and were farmers (61.5%)and had low monthly household incomes. Male patients aged 65 years or younger felt obligated to support themselves, and their family members relied on their limited income, This is due to the traditional Chinese view of men as career-oriented and providing a pivotal part of family income (Su et al., 2019; Wu et al., 2022), therefore, men had experienced higher economic burdens and lower levels of resilience. Patients aged 71 years and older were more likely to have multimorbidity (Lenti et al., 2022), and their physical function, physical activity, psychological state, and quality of life were all lower (Seong et al., 2022). While, this finding is at odds with earlier research, which indicated neither an upgrading in resilience with age (Harms et al., 2019) nor a decrease with age (Tamura et al., 2021; Musich et al., 2022). Age and resilience may not have a simple linear relationship, but rather one that is nuanced. Future research on resilience in older adult patients could take into account various sociocultural and ethnic backgrounds or incorporate a longitudinal design.

The scores of core symptoms and symptom-related interference in the high strength and striving class were substantially lower than those of the remaining two classes. These findings corroborated prior studies that resilience could alleviate symptoms and symptom-related interference (Luo et al., 2020; Tamura et al., 2021). These results also confirmed that improving resilience may be an important strategy for improving the living quality of grown cancer patients. Despite the differences in resilience characteristics among the three classes, there was no noticeable variation in the severity of gastrointestinal cancer-specific symptom ratings. On the one hand, this might be because we investigated patients after esophagectomy who stayed in the hospital for 5–10 days, and gastrointestinal cancer-specific symptoms were prevalent (Wikman et al., 2014; Gupta et al., 2020); on the other hand, such findings highlight the value of precision nursing (Keim-Malpass and Kausch, 2023). To achieve superior therapeutic outcomes, accurate interventions on the basis of various resilience categories should be created and implemented for patients who have the same type or a severity of gastrointestinal cancer-specific symptoms.

### Innovations

This study was designed to categorize resilience by employing LCA, an approach that is unique in that it accounts for individual differences in patients. Using a combination of both items and dimensions for naming enables a comprehensive characterization of resilience performance. Currently, the LCA of resilience has been widely applied to different cancer types, and then different cancer patients have dissimilar treatment modalities, symptom experiences, recovery processes, and clinical outcomes, so specific resilience manifestations also have variability, and the present study applies this type of methodology to the esophageal cancer population to add to the research in this area. In parallel, there are differences in symptoms and symptom distress reported by esophageal cancer patients with diverse resilience categories, which are not distinguished in gastrointestinal-specific panels. In the future, we can target this characteristic to provide precise interventions for patients with distinct resilience categories, which will be beneficial for later patient symptom management and enhance patient outcomes.

### Limitations and future research

There are some limitations to the current investigation. The use of convenience sampling was one drawback of our study, as it might limit the applicability of our discoveries. Furthermore, the cross-sectional design prevented us from observing variations in the memberships of resilience in the patients after esophagectomy and investigating the causal links between resilience and patient-reported symptoms. Longitudinal surveys are needed to determine whether if resilience predicts patient-reported symptoms in patients after esophagectomy.

## Data availability statement

The raw data supporting the conclusions of this article will be made available by the authors, without undue reservation.

## Ethics statement

The studies involving humans were approved by scrutinized and authorized by the Biomedical Ethics Committee of Anhui Medical University (Ref no. 2021H011). The attendees supplied their written informed permission to access this study. The studies were conducted in accordance with the local legislation and institutional requirements. The participants provided their written informed consent to participate in this study.

## Author contributions

SL contributed to conceptualizing and planning the study. YL and ZZ arranged data collecting and collecting measurements. All analyses were performed by YL and confirmed by XZ. XM summarized findings and synthesized content. The final manuscript was revised and prepared for submission by SL, YL, and XM. The article’s presentation was examined and authorized by all authors. All authors contributed to the article and approved the submitted version.
